# Tris(dibenzoyl­methanido-κ^2^ *O*,*O*′)[(6*R*,8*R*)-(−)-7,7-dimethyl-3-(2-pyrid­yl)-5,6,7,8-tetra­hydro-6,8-methano­isoquinoline-κ^2^ *N*,*N*′]terbium(III)

**DOI:** 10.1107/S1600536809019783

**Published:** 2009-06-06

**Authors:** Lei-Qi Chen, Jian-Nan Guo, Wei-Min Xuan, Yi-Ji Lin, Hui Zhang

**Affiliations:** a Department of Chemistry, College of Chemistry and Chemical Engineering, Xiamen University, Xiamen 361005, People’s Republic of China; b The Key Laboratory for Chemical Biology of Fujian Province, Department of Chemistry, College of Chemistry and Chemical Engineering, Xiamen University, Xiamen 361005, People’s Republic of China

## Abstract

In the title compound, [Tb(C_15_H_11_O_2_)_3_(C_17_H_18_N_2_)], the 7,7-dimethyl-3-(2-pyrid­yl)-5,6,7,8-tetra­hydro-6,8-methano­iso­quin­oline (*L^RR^*) ligand coordinates to Tb^III^ through the two N atoms of the heterocycle. The metal centre is also chelated by three deprotonated 1,3-diphenyl­propane-1,3-dione (dbm) ligands, forming enanti­omerically pure [Tb(dbm)_3_ *L^RR^*]. The Tb^III^ atom is located in a distorted square anti­prism of eight coordinating atoms (six O and two N atoms).

## Related literature

For a general background to lanthanide complexes, see: Aspinall (2002 ▶); Li, Chen *et al.* (2007 ▶); Li & Zhang (2008 ▶). For a related structure, see: Li, Zheng *et al.* (2007 ▶). For the synthesis, see: Hayoz *et al.* (1993 ▶); Lennartson *et al.* (2005 ▶).
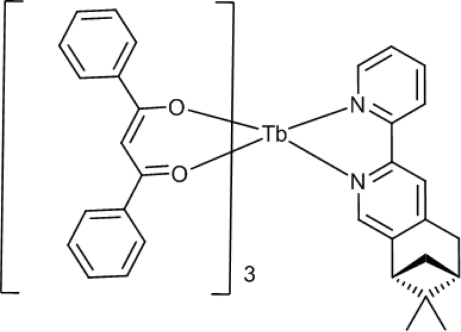


## Experimental

### 

#### Crystal data

[Tb(C_15_H_11_O_2_)_3_(C_17_H_18_N_2_)]
*M* *_r_* = 1078.97
Monoclinic, 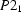

*a* = 9.5158 (19) Å
*b* = 20.790 (4) Å
*c* = 12.769 (3) Å
β = 92.47 (3)°
*V* = 2523.7 (9) Å^3^
*Z* = 2
Mo *K*α radiation
μ = 1.46 mm^−1^
*T* = 296 K
0.51 × 0.43 × 0.34 mm

#### Data collection

Rigaku R-AXIS SPIDER IP diffractometer
Absorption correction: multi-scan (*ABSCOR*; Higashi, 1995 ▶) *T*_min_ = 0.524, *T*_max_ = 0.637
19632 measured reflections
8346 independent reflections
5624 reflections with *I* > 2σ(*I*)
*R*_int_ = 0.090

#### Refinement

*R*[*F* ^2^ > 2σ(*F* ^2^)] = 0.050
*wR*(*F* ^2^) = 0.093
*S* = 0.95
8346 reflections
640 parameters
1 restraint
H-atom parameters constrained
Δρ_max_ = 1.04 e Å^−3^
Δρ_min_ = −1.22 e Å^−3^
Absolute structure: Flack (1983 ▶), 3779 Friedel pairs
Flack parameter: −0.008 (14)

### 

Data collection: *PROCESS-AUTO* (Rigaku, 1998 ▶); cell refinement: *PROCESS-AUTO*; data reduction: *CrystalStructure* (Rigaku/MSC, 2002 ▶); program(s) used to solve structure: *SHELXS97* (Sheldrick, 2008 ▶); program(s) used to refine structure: *SHELXL97* (Sheldrick, 2008 ▶); molecular graphics: *ORTEP-3* (Farrugia, 1997 ▶); software used to prepare material for publication: *SHELXL97*.

## Supplementary Material

Crystal structure: contains datablocks I, global. DOI: 10.1107/S1600536809019783/ng2576sup1.cif

Structure factors: contains datablocks I. DOI: 10.1107/S1600536809019783/ng2576Isup2.hkl

Additional supplementary materials:  crystallographic information; 3D view; checkCIF report

## Figures and Tables

**Table 1:** Selected geometric parameters (Å, °)

N1—Tb1	2.589 (6)
Tb1—O2	2.328 (9)
Tb1—O3	2.312 (7)
Tb1—O5	2.341 (7)
Tb1—O4	2.353 (4)
Tb1—O6	2.372 (7)
Tb1—N2	2.569 (6)

**Fig. 1.**
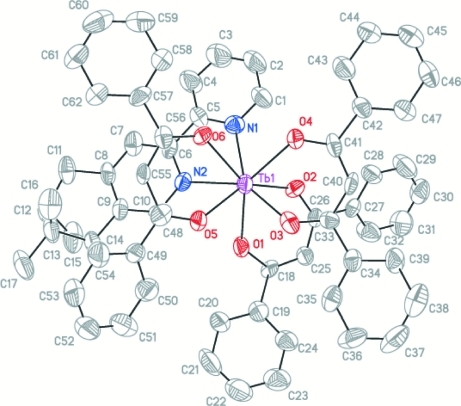
The structure of the title compound, showing the atom-numbering scheme and displacement ellipsoids at the 50% probability level. The H atoms have been omited for charity.

## supplementary crystallographic information

### Comment

Due to the potential applications as luminescence, ferroelectric material and NMR shift reagents, the stereoselective synthesis and characterization of labile lanthanide β-diketonate complexes have obtained a great deal of attentions (Aspinall, 2002; Li, Chen *et al.*, 2007); Li & Zhang, 2008). Herein, we report a chiral complex, [Tb(dbm)_3_*L^RR^*] (Fig. 1), which is isostructural with the previously reported [Eu(dbm)_3_*L^RR^*] (Li, Zheng *et al.*, 2007). For the intrducing of chiral ligand *L^RR^*, the complex was obtained as an enatiopure compound and crystallized in a chiral space group *P*2_1_. The title compound, was synthesized under mild condition. *L^RR^* is introduced as a second ligand, which coordinates to Tb^III^ with three dbm to form enantiomerically pure complex [Tb(dbm)_3_*L^RR^*]. The absolute configuration of the stereocenters in the ligand *L^RR^* remains unchanged during the synthetic procedure.

As indicated in Fig. 1, six O atoms and two N atoms come from the three diketone anions and a chiral 2,2-bipyridine derivative ligand, *L^RR^*, coordinate to a Tb^III^ cation to form a mononuclear neutral eight-coordinated Tb^III^-based complex. In the distorted square antiprism around the Tb^III^ cation, O1, O2, N1, N2 and O3, O4, O5, O6 compose the top and bottom planes of the antiprism. The mean deviations from the two planes are 0.122 and 0.069 Å, and their dihedral angle is 1.83 (1)°. And the top plane is rotated by 37° relative to the bottom plane, which is smaller than 45° for a regular square antipism.

### Experimental

The title compound was prepared *via* a modification of a previously reported method (Hayoz *et al.*, 1993; Lennartson *et al.*, 2005). The *L^RR^* ligand and [Tb(dbm)_3_H_2_O] was synthesized according to reported procedures.

A solution of [Tb(dbm)_3_H_2_O] (15 mg, 0.0178 mmol) in acetone (2 ml) was combined with a solution of (*8R,10R*)-(-)-[4,5]-pineno-2,2'-bipyridine (4.45 mg, 0.0178 mmol) in ethanol (2 ml). And the mixture was stand at room temperature for two days. Yellow block crystals of the title complex were obtained in 75% yield.

### Refinement

The hydrogen atoms were positioned geometrically (C—H = 0.93, 0.98, 0.97 or 0.96Å for phenyl, tertiary, methylene or methyl H atoms respectively) and were included in the refinement in the riding model approximation. The displacement parameters of methyl H atoms were set to 1.5*U*_eq_(C), while those of other H atoms were set to 1.2*U*_eq_(C).

### Crystal data

**Table:**

[Tb(C_15_H_11_O_2_)_3_(C_17_H_18_N_2_)]	*F*(000) = 1100
*M**_r_* = 1078.97	*D*_x_ = 1.420 Mg m^−^^3^
Monoclinic, *P*2_1_	Mo *K*α radiation, λ = 0.71073 Å
Hall symbol: P 2yb	Cell parameters from 11553 reflections
*a* = 9.5158 (19) Å	θ = 3.2–27.5°
*b* = 20.790 (4) Å	µ = 1.46 mm^−^^1^
*c* = 12.769 (3) Å	*T* = 296 K
β = 92.47 (3)°	Block, yellow
*V* = 2523.7 (9) Å^3^	0.51 × 0.43 × 0.34 mm
*Z* = 2	

### Data collection

**Table:**

Rigaku R-AXIS SPIDER IP diffractometer	8346 independent reflections
Radiation source: fine-focus sealed tube	5624 reflections with *I* > 2σ(*I*)
graphite	*R*_int_ = 0.090
ω scans	θ_max_ = 25.0°, θ_min_ = 3.2°
Absorption correction: multi-scan (*ABSCOR*; Higashi, 1995)	*h* = −11→11
*T*_min_ = 0.524, *T*_max_ = 0.637	*k* = −23→24
19632 measured reflections	*l* = −14→15

### Refinement

**Table:**

Refinement on *F*^2^	Secondary atom site location: difference Fourier map
Least-squares matrix: full	Hydrogen site location: inferred from neighbouring sites
*R*[*F*^2^ > 2σ(*F*^2^)] = 0.050	H-atom parameters constrained
*wR*(*F*^2^) = 0.093	*w* = 1/[σ^2^(*F*_o_^2^) + (0.0094*P*)^2^ + 1.367*P*] where *P* = (*F*_o_^2^ + 2*F*_c_^2^)/3
*S* = 0.95	(Δ/σ)_max_ = 0.003
8346 reflections	Δρ_max_ = 1.03 e Å^−^^3^
640 parameters	Δρ_min_ = −1.22 e Å^−^^3^
1 restraint	Absolute structure: Flack (1983), 3779 Friedel pairs
Primary atom site location: structure-invariant direct methods	Flack parameter: −0.008 (14)

### Special details

**Table:**

Geometry. All e.s.d.'s (except the e.s.d. in the dihedral angle between two l.s. planes) are estimated using the full covariance matrix. The cell e.s.d.'s are taken into account individually in the estimation of e.s.d.'s in distances, angles and torsion angles; correlations between e.s.d.'s in cell parameters are only used when they are defined by crystal symmetry. An approximate (isotropic) treatment of cell e.s.d.'s is used for estimating e.s.d.'s involving l.s. planes.
Refinement. Refinement of *F*^2^ against ALL reflections. The weighted *R*-factor *wR* and goodness of fit *S* are based on *F*^2^, conventional *R*-factors *R* are based on *F*, with *F* set to zero for negative *F*^2^. The threshold expression of *F*^2^ > σ(*F*^2^) is used only for calculating *R*-factors(gt) *etc*. and is not relevant to the choice of reflections for refinement. *R*-factors based on *F*^2^ are statistically about twice as large as those based on *F*, and *R*- factors based on ALL data will be even larger.

### Fractional atomic coordinates and isotropic or equivalent isotropic displacement parameters (Å^2^)

**Table:**

	*x*	*y*	*z*	*U*_iso_*/*U*_eq_	
C1	0.3453 (11)	0.4955 (5)	1.0559 (7)	0.061 (4)	
H1A	0.2627	0.4725	1.0640	0.073*	
N1	0.3795 (6)	0.5115 (7)	0.9587 (4)	0.0455 (15)	
O1	0.2344 (7)	0.4463 (3)	0.6603 (6)	0.044 (2)	
Tb1	0.19194 (4)	0.51094 (3)	0.80567 (3)	0.03880 (11)	
C2	0.4251 (12)	0.5109 (10)	1.1437 (7)	0.085 (3)	
H2A	0.3963	0.5000	1.2101	0.102*	
N2	0.4480 (7)	0.5358 (3)	0.7640 (6)	0.0378 (19)	
O2	0.1996 (9)	0.4047 (4)	0.8634 (8)	0.049 (3)	
C3	0.5473 (15)	0.5427 (6)	1.1313 (10)	0.099 (5)	
H3A	0.6048	0.5527	1.1897	0.118*	
O3	−0.0322 (7)	0.4832 (2)	0.7448 (5)	0.0479 (17)	
C4	0.5867 (12)	0.5599 (5)	1.0347 (9)	0.076 (3)	
H4A	0.6697	0.5827	1.0266	0.091*	
O4	0.0459 (5)	0.5113 (5)	0.9497 (3)	0.0460 (13)	
C5	0.5009 (11)	0.5429 (4)	0.9467 (7)	0.049 (3)	
O5	0.1579 (7)	0.5793 (3)	0.6611 (6)	0.0403 (18)	
C6	0.5388 (9)	0.5584 (4)	0.8388 (8)	0.040 (2)	
O6	0.2217 (10)	0.6188 (3)	0.8630 (8)	0.047 (3)	
C7	0.6570 (10)	0.5914 (4)	0.8162 (9)	0.056 (3)	
H7A	0.7162	0.6067	0.8705	0.068*	
C8	0.6903 (13)	0.6027 (5)	0.7130 (10)	0.046 (3)	
C9	0.6051 (10)	0.5767 (4)	0.6343 (8)	0.047 (3)	
C10	0.4818 (10)	0.5450 (3)	0.6637 (7)	0.041 (2)	
H10A	0.4204	0.5295	0.6108	0.049*	
C11	0.8151 (11)	0.6428 (5)	0.6816 (10)	0.062 (3)	
H11A	0.9016	0.6237	0.7100	0.075*	
H11B	0.8077	0.6859	0.7100	0.075*	
C12	0.8186 (12)	0.6461 (5)	0.5617 (10)	0.060 (3)	
H12A	0.8977	0.6705	0.5352	0.072*	
C13	0.6755 (16)	0.6621 (6)	0.5090 (11)	0.065 (4)	
C14	0.6401 (12)	0.5886 (4)	0.5235 (8)	0.058 (3)	
H14A	0.5803	0.5681	0.4687	0.070*	
C15	0.8052 (12)	0.5770 (5)	0.5200 (10)	0.083 (4)	
H15A	0.8416	0.5443	0.5681	0.099*	
H15B	0.8392	0.5710	0.4501	0.099*	
C16	0.5843 (11)	0.7116 (5)	0.5623 (9)	0.069 (3)	
H16A	0.6169	0.7541	0.5467	0.104*	
H16B	0.5903	0.7049	0.6367	0.104*	
H16C	0.4884	0.7070	0.5370	0.104*	
C17	0.6831 (14)	0.6796 (6)	0.3934 (9)	0.104 (5)	
H17A	0.7039	0.7246	0.3870	0.155*	
H17B	0.5945	0.6705	0.3578	0.155*	
H17C	0.7558	0.6548	0.3626	0.155*	
C18	0.1838 (15)	0.3886 (6)	0.6372 (12)	0.047 (4)	
C19	0.1776 (16)	0.3760 (6)	0.5217 (13)	0.050 (4)	
C20	0.2664 (15)	0.4081 (6)	0.4562 (11)	0.067 (4)	
H20A	0.3296	0.4386	0.4832	0.080*	
C21	0.260 (2)	0.3937 (7)	0.3468 (14)	0.100 (7)	
H21A	0.3191	0.4149	0.3021	0.120*	
C22	0.166 (2)	0.3486 (9)	0.3073 (14)	0.094 (7)	
H22A	0.1597	0.3404	0.2357	0.113*	
C23	0.0827 (14)	0.3161 (5)	0.3729 (10)	0.076 (4)	
H23A	0.0206	0.2850	0.3463	0.091*	
C24	0.0900 (13)	0.3290 (4)	0.4782 (8)	0.059 (3)	
H24A	0.0340	0.3054	0.5221	0.070*	
C25	0.1452 (11)	0.3446 (4)	0.7094 (7)	0.043 (3)	
H25A	0.1067	0.3061	0.6846	0.052*	
C26	0.1589 (10)	0.3527 (4)	0.8162 (7)	0.037 (2)	
C27	0.1249 (9)	0.2964 (3)	0.8876 (7)	0.038 (2)	
C28	0.0840 (12)	0.3068 (4)	0.9875 (7)	0.062 (3)	
H28A	0.0795	0.3485	1.0133	0.074*	
C29	0.0499 (13)	0.2565 (5)	1.0495 (8)	0.085 (4)	
H29A	0.0217	0.2645	1.1171	0.102*	
C30	0.0559 (12)	0.1946 (4)	1.0152 (9)	0.067 (3)	
H30A	0.0297	0.1610	1.0583	0.080*	
C31	0.1002 (13)	0.1823 (5)	0.9180 (10)	0.079 (4)	
H31A	0.1089	0.1403	0.8944	0.095*	
C32	0.1324 (13)	0.2336 (4)	0.8546 (8)	0.073 (4)	
H32A	0.1602	0.2254	0.7869	0.087*	
C33	−0.1299 (10)	0.4515 (4)	0.7860 (7)	0.038 (2)	
C34	−0.2340 (12)	0.4184 (5)	0.7124 (9)	0.043 (3)	
C35	−0.2038 (11)	0.4120 (4)	0.6077 (10)	0.052 (3)	
H35A	−0.1190	0.4277	0.5845	0.062*	
C36	−0.2997 (17)	0.3822 (6)	0.5360 (12)	0.074 (5)	
H36A	−0.2799	0.3795	0.4654	0.089*	
C37	−0.4207 (15)	0.3576 (6)	0.5704 (12)	0.085 (4)	
H37A	−0.4816	0.3355	0.5243	0.101*	
C38	−0.4543 (12)	0.3653 (5)	0.6740 (12)	0.076 (4)	
H38A	−0.5396	0.3496	0.6963	0.091*	
C39	−0.3618 (11)	0.3964 (5)	0.7463 (9)	0.052 (3)	
H39A	−0.3859	0.4022	0.8154	0.063*	
C40	−0.1415 (10)	0.4455 (4)	0.8923 (8)	0.046 (2)	
H40A	−0.2116	0.4184	0.9152	0.055*	
C41	−0.0589 (10)	0.4760 (4)	0.9682 (7)	0.042 (2)	
C42	−0.0893 (10)	0.4674 (4)	1.0826 (7)	0.048 (2)	
C43	−0.0303 (9)	0.5112 (9)	1.1547 (6)	0.061 (2)	
H43A	0.0294	0.5434	1.1326	0.073*	
C44	−0.0616 (11)	0.5064 (9)	1.2606 (7)	0.080 (3)	
H44A	−0.0241	0.5360	1.3086	0.096*	
C45	−0.1463 (15)	0.4586 (6)	1.2936 (9)	0.094 (4)	
H45A	−0.1647	0.4553	1.3643	0.112*	
C46	−0.2038 (14)	0.4162 (6)	1.2253 (9)	0.089 (4)	
H46A	−0.2642	0.3848	1.2492	0.107*	
C47	−0.1749 (13)	0.4182 (5)	1.1191 (8)	0.075 (4)	
H47A	−0.2119	0.3872	1.0732	0.089*	
C48	0.2014 (13)	0.6338 (6)	0.6362 (12)	0.039 (4)	
C49	0.1978 (14)	0.6503 (6)	0.5226 (13)	0.041 (4)	
C50	0.2037 (14)	0.5989 (6)	0.4524 (12)	0.061 (4)	
H50A	0.2033	0.5570	0.4777	0.073*	
C51	0.2099 (19)	0.6093 (8)	0.3490 (14)	0.085 (5)	
H51A	0.2173	0.5745	0.3037	0.103*	
C52	0.2054 (16)	0.6708 (10)	0.3091 (14)	0.081 (6)	
H52A	0.2059	0.6774	0.2371	0.097*	
C53	0.2004 (14)	0.7212 (5)	0.3744 (10)	0.073 (4)	
H53A	0.1987	0.7628	0.3477	0.088*	
C54	0.1977 (12)	0.7112 (5)	0.4813 (9)	0.061 (3)	
H54A	0.1957	0.7464	0.5261	0.073*	
C55	0.2517 (10)	0.6806 (4)	0.7104 (7)	0.039 (2)	
H55A	0.2743	0.7212	0.6857	0.047*	
C56	0.2688 (11)	0.6690 (4)	0.8176 (8)	0.041 (2)	
C57	0.3457 (10)	0.7162 (4)	0.8887 (8)	0.049 (3)	
C58	0.3327 (13)	0.7120 (5)	0.9947 (9)	0.077 (4)	
H58A	0.2728	0.6816	1.0221	0.092*	
C59	0.4101 (18)	0.7536 (8)	1.0620 (12)	0.106 (5)	
H59A	0.4028	0.7504	1.1343	0.127*	
C60	0.4949 (13)	0.7981 (6)	1.0211 (12)	0.082 (4)	
H60A	0.5484	0.8248	1.0657	0.098*	
C61	0.5031 (16)	0.8045 (6)	0.9181 (12)	0.089 (5)	
H61A	0.5586	0.8369	0.8913	0.106*	
C62	0.4301 (12)	0.7636 (4)	0.8507 (9)	0.072 (3)	
H62B	0.4381	0.7680	0.7787	0.087*	

### Atomic displacement parameters (Å^2^)

**Table:**

	*U*^11^	*U*^22^	*U*^33^	*U*^12^	*U*^13^	*U*^23^
C1	0.073 (8)	0.068 (10)	0.041 (6)	−0.002 (5)	0.011 (5)	0.009 (5)
N1	0.046 (4)	0.046 (3)	0.045 (4)	0.001 (8)	0.006 (3)	−0.008 (9)
O1	0.039 (5)	0.045 (4)	0.049 (4)	−0.009 (3)	0.016 (4)	0.001 (3)
Tb1	0.0427 (2)	0.03169 (16)	0.0427 (2)	−0.0053 (4)	0.01018 (15)	0.0003 (4)
C2	0.100 (9)	0.116 (8)	0.039 (6)	−0.024 (15)	0.003 (6)	0.013 (15)
N2	0.028 (5)	0.037 (4)	0.050 (5)	−0.007 (3)	0.010 (4)	−0.007 (3)
O2	0.051 (6)	0.047 (5)	0.049 (7)	−0.014 (4)	0.002 (5)	−0.013 (4)
C3	0.097 (12)	0.143 (12)	0.054 (9)	−0.036 (9)	−0.010 (8)	0.006 (7)
O3	0.054 (5)	0.049 (3)	0.042 (4)	−0.001 (3)	0.014 (3)	0.007 (3)
C4	0.058 (9)	0.122 (9)	0.048 (8)	−0.019 (7)	−0.001 (6)	0.007 (7)
O4	0.046 (3)	0.050 (3)	0.043 (3)	−0.015 (6)	0.014 (3)	−0.004 (7)
C5	0.050 (7)	0.057 (5)	0.042 (6)	−0.001 (5)	0.012 (5)	0.008 (4)
O5	0.045 (5)	0.034 (3)	0.042 (4)	−0.009 (3)	0.002 (4)	0.002 (3)
C6	0.026 (6)	0.035 (4)	0.060 (7)	−0.004 (4)	0.006 (5)	0.003 (4)
O6	0.072 (7)	0.024 (4)	0.047 (7)	−0.011 (4)	0.014 (5)	−0.015 (4)
C7	0.037 (7)	0.062 (6)	0.069 (8)	−0.007 (5)	−0.014 (6)	0.021 (6)
C8	0.031 (8)	0.053 (6)	0.055 (8)	−0.010 (6)	0.022 (7)	0.008 (5)
C9	0.048 (7)	0.033 (5)	0.063 (7)	−0.005 (4)	0.022 (6)	−0.006 (4)
C10	0.048 (7)	0.042 (4)	0.034 (6)	−0.006 (4)	0.007 (5)	−0.014 (4)
C11	0.033 (6)	0.062 (6)	0.091 (10)	−0.004 (5)	0.004 (6)	0.031 (7)
C12	0.041 (8)	0.052 (7)	0.089 (10)	0.000 (5)	0.026 (7)	0.007 (6)
C13	0.078 (10)	0.048 (7)	0.070 (10)	−0.013 (6)	0.035 (8)	0.010 (6)
C14	0.076 (9)	0.054 (6)	0.047 (7)	−0.022 (5)	0.023 (6)	−0.001 (5)
C15	0.076 (10)	0.058 (7)	0.117 (11)	0.005 (6)	0.050 (9)	0.003 (7)
C16	0.047 (7)	0.059 (6)	0.102 (10)	0.012 (5)	0.002 (7)	0.024 (7)
C17	0.117 (12)	0.122 (10)	0.073 (10)	−0.041 (9)	0.015 (9)	0.038 (8)
C18	0.067 (11)	0.020 (7)	0.055 (10)	−0.011 (5)	0.015 (8)	−0.023 (6)
C19	0.067 (11)	0.037 (8)	0.048 (10)	0.004 (6)	0.014 (8)	−0.006 (7)
C20	0.096 (12)	0.057 (7)	0.047 (8)	−0.020 (7)	0.003 (8)	0.007 (6)
C21	0.18 (2)	0.076 (11)	0.052 (11)	−0.023 (11)	0.040 (11)	0.014 (8)
C22	0.156 (18)	0.075 (12)	0.052 (13)	−0.010 (10)	0.002 (12)	0.007 (8)
C23	0.094 (12)	0.070 (8)	0.063 (9)	0.004 (7)	0.004 (8)	−0.016 (6)
C24	0.086 (10)	0.044 (6)	0.047 (7)	0.000 (6)	0.012 (7)	−0.006 (5)
C25	0.060 (8)	0.033 (5)	0.039 (6)	−0.004 (5)	0.015 (6)	−0.004 (4)
C26	0.036 (7)	0.028 (4)	0.048 (6)	−0.005 (4)	0.001 (5)	0.007 (4)
C27	0.040 (6)	0.026 (4)	0.049 (6)	−0.005 (4)	0.003 (5)	−0.002 (4)
C28	0.092 (10)	0.053 (6)	0.041 (7)	−0.013 (6)	0.011 (6)	0.003 (5)
C29	0.119 (13)	0.081 (8)	0.057 (8)	−0.003 (7)	0.034 (8)	0.006 (7)
C30	0.075 (9)	0.048 (6)	0.077 (9)	−0.014 (5)	−0.009 (7)	0.023 (6)
C31	0.111 (11)	0.044 (6)	0.082 (9)	−0.006 (6)	0.003 (8)	0.013 (6)
C32	0.118 (11)	0.049 (6)	0.052 (7)	0.004 (6)	0.017 (7)	−0.003 (5)
C33	0.046 (7)	0.040 (5)	0.029 (5)	0.004 (4)	−0.001 (5)	0.000 (4)
C34	0.041 (8)	0.037 (5)	0.052 (7)	−0.003 (5)	0.007 (6)	0.003 (4)
C35	0.048 (7)	0.032 (5)	0.074 (9)	0.000 (4)	−0.005 (6)	0.010 (6)
C36	0.092 (13)	0.064 (10)	0.063 (10)	−0.011 (8)	−0.021 (8)	−0.016 (7)
C37	0.071 (11)	0.071 (8)	0.108 (13)	−0.023 (7)	−0.043 (9)	−0.003 (8)
C38	0.043 (8)	0.063 (7)	0.120 (12)	−0.008 (5)	−0.005 (8)	0.008 (7)
C39	0.028 (7)	0.075 (7)	0.054 (8)	−0.007 (5)	0.001 (6)	0.012 (6)
C40	0.034 (6)	0.046 (5)	0.057 (7)	−0.021 (4)	0.004 (5)	0.003 (4)
C41	0.034 (6)	0.042 (5)	0.052 (6)	0.000 (4)	0.024 (5)	0.008 (4)
C42	0.042 (7)	0.057 (5)	0.044 (6)	0.002 (4)	0.012 (5)	−0.001 (5)
C43	0.077 (6)	0.057 (4)	0.049 (5)	0.002 (12)	0.016 (5)	0.009 (12)
C44	0.114 (9)	0.082 (7)	0.046 (6)	−0.029 (12)	0.018 (6)	−0.010 (10)
C45	0.127 (13)	0.113 (10)	0.042 (8)	−0.036 (9)	0.020 (8)	−0.005 (7)
C46	0.114 (12)	0.103 (9)	0.052 (8)	−0.041 (8)	0.026 (8)	0.014 (6)
C47	0.100 (10)	0.075 (7)	0.051 (7)	−0.028 (6)	0.025 (7)	−0.014 (5)
C48	0.015 (7)	0.046 (8)	0.054 (10)	0.005 (5)	0.000 (6)	−0.008 (7)
C49	0.033 (8)	0.046 (8)	0.043 (10)	−0.005 (6)	−0.002 (7)	−0.004 (7)
C50	0.067 (10)	0.055 (7)	0.060 (9)	−0.002 (6)	−0.008 (7)	0.003 (6)
C51	0.123 (14)	0.083 (10)	0.051 (10)	−0.009 (9)	0.005 (9)	0.003 (7)
C52	0.083 (13)	0.114 (17)	0.047 (12)	−0.029 (10)	0.007 (9)	0.004 (10)
C53	0.096 (11)	0.062 (7)	0.063 (9)	−0.019 (7)	−0.002 (8)	0.023 (6)
C54	0.071 (9)	0.049 (6)	0.064 (8)	−0.007 (6)	0.010 (7)	0.004 (5)
C55	0.049 (7)	0.025 (4)	0.043 (6)	−0.009 (4)	0.008 (5)	0.006 (4)
C56	0.041 (7)	0.029 (5)	0.055 (7)	0.002 (4)	0.012 (5)	0.006 (4)
C57	0.055 (7)	0.037 (5)	0.055 (7)	−0.001 (4)	0.005 (5)	−0.015 (4)
C58	0.104 (11)	0.070 (7)	0.057 (8)	−0.039 (7)	0.010 (7)	−0.006 (6)
C59	0.130 (16)	0.131 (14)	0.056 (9)	−0.052 (11)	0.006 (10)	−0.028 (9)
C60	0.067 (10)	0.089 (9)	0.090 (11)	−0.009 (7)	−0.008 (8)	−0.039 (8)
C61	0.104 (13)	0.078 (9)	0.085 (12)	−0.046 (8)	0.016 (10)	−0.027 (8)
C62	0.093 (10)	0.063 (6)	0.061 (8)	−0.033 (6)	0.007 (7)	−0.013 (6)

### Geometric parameters (Å, °)

**Table:**

C1—N1	1.338 (10)	C25—H25A	0.9300
C1—C2	1.365 (13)	C26—C27	1.525 (10)
C1—H1A	0.9300	C27—C28	1.367 (11)
N1—C5	1.342 (12)	C27—C32	1.375 (11)
N1—Tb1	2.589 (6)	C28—C29	1.360 (12)
O1—C18	1.321 (13)	C28—H28A	0.9300
O1—Tb1	2.341 (7)	C29—C30	1.360 (13)
Tb1—O2	2.328 (9)	C29—H29A	0.9300
Tb1—O3	2.312 (7)	C30—C31	1.352 (14)
Tb1—O5	2.341 (7)	C30—H30A	0.9300
Tb1—O4	2.353 (4)	C31—C32	1.382 (12)
Tb1—O6	2.372 (7)	C31—H31A	0.9300
Tb1—N2	2.569 (6)	C32—H32A	0.9300
C2—C3	1.352 (16)	C33—C40	1.372 (11)
C2—H2A	0.9300	C33—C34	1.503 (14)
N2—C6	1.344 (11)	C34—C35	1.385 (16)
N2—C10	1.348 (10)	C34—C39	1.386 (13)
O2—C26	1.290 (11)	C35—C36	1.409 (16)
C3—C4	1.353 (14)	C35—H35A	0.9300
C3—H3A	0.9300	C36—C37	1.350 (18)
O3—C33	1.272 (9)	C36—H36A	0.9300
C4—C5	1.405 (14)	C37—C38	1.384 (16)
C4—H4A	0.9300	C37—H37A	0.9300
O4—C41	1.267 (10)	C38—C39	1.405 (15)
C5—C6	1.475 (12)	C38—H38A	0.9300
O5—C48	1.253 (14)	C39—H39A	0.9300
C6—C7	1.360 (11)	C40—C41	1.376 (12)
O6—C56	1.284 (11)	C40—H40A	0.9300
C7—C8	1.388 (15)	C41—C42	1.513 (12)
C7—H7A	0.9300	C42—C43	1.395 (16)
C8—C9	1.374 (15)	C42—C47	1.401 (12)
C8—C11	1.519 (13)	C43—C44	1.400 (10)
C9—C10	1.411 (11)	C43—H43A	0.9300
C9—C14	1.489 (12)	C44—C45	1.357 (18)
C10—H10A	0.9300	C44—H44A	0.9300
C11—C12	1.533 (16)	C45—C46	1.340 (15)
C11—H11A	0.9700	C45—H45A	0.9300
C11—H11B	0.9700	C46—C47	1.396 (13)
C12—C13	1.530 (18)	C46—H46A	0.9300
C12—C15	1.535 (13)	C47—H47A	0.9300
C12—H12A	0.9800	C48—C55	1.425 (16)
C13—C16	1.524 (16)	C48—C49	1.49 (2)
C13—C17	1.526 (16)	C49—C54	1.372 (16)
C13—C14	1.577 (13)	C49—C50	1.397 (18)
C14—C15	1.592 (15)	C50—C51	1.34 (2)
C14—H14A	0.9800	C50—H50A	0.9300
C15—H15A	0.9700	C51—C52	1.38 (2)
C15—H15B	0.9700	C51—H51A	0.9300
C16—H16A	0.9600	C52—C53	1.34 (2)
C16—H16B	0.9600	C52—H52A	0.9300
C16—H16C	0.9600	C53—C54	1.382 (14)
C17—H17A	0.9600	C53—H53A	0.9300
C17—H17B	0.9600	C54—H54A	0.9300
C17—H17C	0.9600	C55—C56	1.393 (12)
C18—C25	1.361 (16)	C55—H55A	0.9300
C18—C19	1.50 (2)	C56—C57	1.506 (12)
C19—C24	1.385 (17)	C57—C58	1.367 (13)
C19—C20	1.385 (17)	C57—C62	1.372 (12)
C20—C21	1.43 (2)	C58—C59	1.406 (16)
C20—H20A	0.9300	C58—H58A	0.9300
C21—C22	1.38 (2)	C59—C60	1.348 (17)
C21—H21A	0.9300	C59—H59A	0.9300
C22—C23	1.358 (19)	C60—C61	1.328 (17)
C22—H22A	0.9300	C60—H60A	0.9300
C23—C24	1.370 (14)	C61—C62	1.377 (15)
C23—H23A	0.9300	C61—H61A	0.9300
C24—H24A	0.9300	C62—H62B	0.9300
C25—C26	1.374 (12)		
			
N1—C1—C2	123.6 (10)	C23—C22—C21	120.0 (17)
N1—C1—H1A	118.2	C23—C22—H22A	120.0
C2—C1—H1A	118.2	C21—C22—H22A	120.0
C5—N1—C1	118.2 (8)	C22—C23—C24	120.1 (14)
C5—N1—Tb1	119.0 (7)	C22—C23—H23A	120.0
C1—N1—Tb1	120.8 (6)	C24—C23—H23A	120.0
C18—O1—Tb1	128.7 (7)	C19—C24—C23	122.4 (12)
O2—Tb1—O3	83.4 (3)	C19—C24—H24A	118.8
O2—Tb1—O5	145.8 (3)	C23—C24—H24A	118.8
O3—Tb1—O5	78.0 (2)	C18—C25—C26	125.0 (9)
O2—Tb1—O1	72.7 (3)	C18—C25—H25A	117.5
O3—Tb1—O1	77.2 (2)	C26—C25—H25A	117.5
O5—Tb1—O1	75.32 (17)	O2—C26—C25	125.4 (8)
O2—Tb1—O4	76.5 (3)	O2—C26—C27	115.5 (9)
O3—Tb1—O4	72.31 (19)	C25—C26—C27	119.0 (8)
O5—Tb1—O4	123.3 (3)	C28—C27—C32	117.1 (8)
O1—Tb1—O4	138.5 (3)	C28—C27—C26	120.9 (7)
O2—Tb1—O6	142.88 (17)	C32—C27—C26	122.0 (8)
O3—Tb1—O6	115.8 (3)	C27—C28—C29	120.5 (9)
O5—Tb1—O6	71.3 (3)	C27—C28—H28A	119.8
O1—Tb1—O6	139.9 (3)	C29—C28—H28A	119.8
O4—Tb1—O6	79.8 (3)	C30—C29—C28	121.7 (10)
O2—Tb1—N2	103.9 (3)	C30—C29—H29A	119.1
O3—Tb1—N2	148.2 (2)	C28—C29—H29A	119.1
O5—Tb1—N2	79.3 (2)	C29—C30—C31	119.5 (9)
O1—Tb1—N2	75.7 (2)	C29—C30—H30A	120.2
O4—Tb1—N2	139.4 (2)	C31—C30—H30A	120.2
O6—Tb1—N2	76.8 (2)	C30—C31—C32	118.6 (10)
O2—Tb1—N1	75.8 (4)	C30—C31—H31A	120.7
O3—Tb1—N1	148.3 (2)	C32—C31—H31A	120.7
O5—Tb1—N1	131.3 (3)	C27—C32—C31	122.5 (10)
O1—Tb1—N1	117.7 (3)	C27—C32—H32A	118.7
O4—Tb1—N1	79.69 (17)	C31—C32—H32A	118.7
O6—Tb1—N1	72.2 (4)	O3—C33—C40	123.2 (9)
N2—Tb1—N1	61.7 (2)	O3—C33—C34	116.8 (8)
C3—C2—C1	118.0 (10)	C40—C33—C34	120.0 (8)
C3—C2—H2A	121.0	C35—C34—C39	119.4 (11)
C1—C2—H2A	121.0	C35—C34—C33	119.3 (9)
C6—N2—C10	117.0 (7)	C39—C34—C33	121.2 (10)
C6—N2—Tb1	120.7 (5)	C34—C35—C36	120.9 (11)
C10—N2—Tb1	119.5 (6)	C34—C35—H35A	119.5
C26—O2—Tb1	130.0 (8)	C36—C35—H35A	119.5
C2—C3—C4	120.7 (12)	C37—C36—C35	119.6 (14)
C2—C3—H3A	119.7	C37—C36—H36A	120.2
C4—C3—H3A	119.7	C35—C36—H36A	120.2
C33—O3—Tb1	132.1 (6)	C36—C37—C38	120.2 (12)
C3—C4—C5	119.2 (11)	C36—C37—H37A	119.9
C3—C4—H4A	120.4	C38—C37—H37A	119.9
C5—C4—H4A	120.4	C37—C38—C39	121.1 (11)
C41—O4—Tb1	129.8 (6)	C37—C38—H38A	119.4
N1—C5—C4	120.3 (8)	C39—C38—H38A	119.4
N1—C5—C6	117.3 (9)	C34—C39—C38	118.7 (11)
C4—C5—C6	122.4 (9)	C34—C39—H39A	120.6
C48—O5—Tb1	135.5 (8)	C38—C39—H39A	120.6
N2—C6—C7	122.6 (9)	C33—C40—C41	126.0 (8)
N2—C6—C5	114.3 (8)	C33—C40—H40A	117.0
C7—C6—C5	123.1 (10)	C41—C40—H40A	117.0
C56—O6—Tb1	132.0 (7)	O4—C41—C40	124.5 (8)
C6—C7—C8	120.8 (11)	O4—C41—C42	115.7 (9)
C6—C7—H7A	119.6	C40—C41—C42	119.9 (8)
C8—C7—H7A	119.6	C43—C42—C47	118.7 (9)
C9—C8—C7	118.4 (9)	C43—C42—C41	118.1 (8)
C9—C8—C11	117.8 (10)	C47—C42—C41	123.1 (9)
C7—C8—C11	123.8 (12)	C42—C43—C44	119.6 (14)
C8—C9—C10	117.5 (9)	C42—C43—H43A	120.2
C8—C9—C14	118.7 (9)	C44—C43—H43A	120.2
C10—C9—C14	123.6 (10)	C45—C44—C43	120.4 (15)
N2—C10—C9	123.6 (9)	C45—C44—H44A	119.8
N2—C10—H10A	118.2	C43—C44—H44A	119.8
C9—C10—H10A	118.2	C46—C45—C44	120.7 (11)
C8—C11—C12	109.8 (10)	C46—C45—H45A	119.6
C8—C11—H11A	109.7	C44—C45—H45A	119.6
C12—C11—H11A	109.7	C45—C46—C47	121.4 (11)
C8—C11—H11B	109.7	C45—C46—H46A	119.3
C12—C11—H11B	109.7	C47—C46—H46A	119.3
H11A—C11—H11B	108.2	C46—C47—C42	119.1 (10)
C13—C12—C11	113.1 (9)	C46—C47—H47A	120.4
C13—C12—C15	89.5 (10)	C42—C47—H47A	120.4
C11—C12—C15	107.4 (9)	O5—C48—C55	123.6 (13)
C13—C12—H12A	114.7	O5—C48—C49	117.4 (12)
C11—C12—H12A	114.7	C55—C48—C49	119.0 (12)
C15—C12—H12A	114.7	C54—C49—C50	117.3 (14)
C16—C13—C17	108.8 (10)	C54—C49—C48	125.8 (13)
C16—C13—C12	117.6 (12)	C50—C49—C48	116.8 (13)
C17—C13—C12	113.5 (10)	C51—C50—C49	120.9 (14)
C16—C13—C14	118.2 (9)	C51—C50—H50A	119.6
C17—C13—C14	111.3 (11)	C49—C50—H50A	119.6
C12—C13—C14	85.9 (9)	C50—C51—C52	120.7 (16)
C9—C14—C13	109.3 (8)	C50—C51—H51A	119.6
C9—C14—C15	105.3 (9)	C52—C51—H51A	119.6
C13—C14—C15	85.9 (8)	C53—C52—C51	119.8 (16)
C9—C14—H14A	117.3	C53—C52—H52A	120.1
C13—C14—H14A	117.3	C51—C52—H52A	120.1
C15—C14—H14A	117.3	C52—C53—C54	120.0 (12)
C12—C15—C14	85.2 (7)	C52—C53—H53A	120.0
C12—C15—H15A	114.5	C54—C53—H53A	120.0
C14—C15—H15A	114.5	C49—C54—C53	121.2 (11)
C12—C15—H15B	114.5	C49—C54—H54A	119.4
C14—C15—H15B	114.5	C53—C54—H54A	119.4
H15A—C15—H15B	111.6	C56—C55—C48	123.8 (9)
C13—C16—H16A	109.5	C56—C55—H55A	118.1
C13—C16—H16B	109.5	C48—C55—H55A	118.1
H16A—C16—H16B	109.5	O6—C56—C55	123.8 (9)
C13—C16—H16C	109.5	O6—C56—C57	115.4 (9)
H16A—C16—H16C	109.5	C55—C56—C57	120.9 (8)
H16B—C16—H16C	109.5	C58—C57—C62	118.4 (9)
C13—C17—H17A	109.5	C58—C57—C56	119.5 (9)
C13—C17—H17B	109.5	C62—C57—C56	122.1 (9)
H17A—C17—H17B	109.5	C57—C58—C59	119.9 (11)
C13—C17—H17C	109.5	C57—C58—H58A	120.1
H17A—C17—H17C	109.5	C59—C58—H58A	120.1
H17B—C17—H17C	109.5	C60—C59—C58	119.5 (13)
O1—C18—C25	124.4 (12)	C60—C59—H59A	120.3
O1—C18—C19	112.2 (12)	C58—C59—H59A	120.3
C25—C18—C19	123.4 (11)	C61—C60—C59	120.9 (13)
C24—C19—C20	118.0 (14)	C61—C60—H60A	119.5
C24—C19—C18	121.0 (13)	C59—C60—H60A	119.5
C20—C19—C18	120.8 (14)	C60—C61—C62	120.5 (12)
C19—C20—C21	119.2 (14)	C60—C61—H61A	119.7
C19—C20—H20A	120.4	C62—C61—H61A	119.7
C21—C20—H20A	120.4	C61—C62—C57	120.6 (11)
C22—C21—C20	120.1 (15)	C61—C62—H62B	119.7
C22—C21—H21A	119.9	C57—C62—H62B	119.7
C20—C21—H21A	119.9		
			
C2—C1—N1—C5	2(2)	C8—C9—C14—C13	−45.9 (14)
C2—C1—N1—Tb1	−161.5 (13)	C10—C9—C14—C13	128.2 (10)
C18—O1—Tb1—O2	36.6 (11)	C8—C9—C14—C15	45.0 (12)
C18—O1—Tb1—O3	−50.4 (11)	C10—C9—C14—C15	−140.9 (9)
C18—O1—Tb1—O5	−131.1 (11)	C16—C13—C14—C9	−41.5 (16)
C18—O1—Tb1—O4	−7.1 (12)	C17—C13—C14—C9	−168.5 (11)
C18—O1—Tb1—O6	−165.4 (10)	C12—C13—C14—C9	77.8 (10)
C18—O1—Tb1—N2	146.4 (11)	C16—C13—C14—C15	−146.2 (13)
C18—O1—Tb1—N1	99.7 (11)	C17—C13—C14—C15	86.7 (11)
C5—N1—Tb1—O2	135.2 (9)	C12—C13—C14—C15	−26.9 (8)
C1—N1—Tb1—O2	−61.4 (10)	C13—C12—C15—C14	−27.7 (8)
C5—N1—Tb1—O3	−174.3 (6)	C11—C12—C15—C14	86.4 (9)
C1—N1—Tb1—O3	−10.8 (14)	C9—C14—C15—C12	−82.0 (8)
C5—N1—Tb1—O5	−20.7 (10)	C13—C14—C15—C12	26.9 (9)
C1—N1—Tb1—O5	142.8 (9)	Tb1—O1—C18—C25	−28.7 (19)
C5—N1—Tb1—O1	73.9 (10)	Tb1—O1—C18—C19	153.1 (9)
C1—N1—Tb1—O1	−122.7 (9)	O1—C18—C19—C24	−159.4 (12)
C5—N1—Tb1—O4	−146.3 (9)	C25—C18—C19—C24	22 (2)
C1—N1—Tb1—O4	17.2 (10)	O1—C18—C19—C20	25 (2)
C5—N1—Tb1—O6	−63.8 (9)	C25—C18—C19—C20	−153.2 (13)
C1—N1—Tb1—O6	99.7 (10)	C24—C19—C20—C21	3(2)
C5—N1—Tb1—N2	20.6 (7)	C18—C19—C20—C21	178.5 (15)
C1—N1—Tb1—N2	−176.0 (12)	C19—C20—C21—C22	0(3)
N1—C1—C2—C3	−2(3)	C20—C21—C22—C23	−2(3)
O2—Tb1—N2—C6	−88.2 (6)	C21—C22—C23—C24	1(3)
O3—Tb1—N2—C6	171.8 (5)	C20—C19—C24—C23	−4(2)
O5—Tb1—N2—C6	126.7 (6)	C18—C19—C24—C23	−179.5 (13)
O1—Tb1—N2—C6	−155.9 (6)	C22—C23—C24—C19	2(2)
O4—Tb1—N2—C6	−2.9 (8)	O1—C18—C25—C26	−3(2)
O6—Tb1—N2—C6	53.6 (6)	C19—C18—C25—C26	175.1 (13)
N1—Tb1—N2—C6	−23.0 (6)	Tb1—O2—C26—C25	23.7 (16)
O2—Tb1—N2—C10	111.7 (5)	Tb1—O2—C26—C27	−156.6 (6)
O3—Tb1—N2—C10	11.6 (7)	C18—C25—C26—O2	5.8 (19)
O5—Tb1—N2—C10	−33.4 (5)	C18—C25—C26—C27	−173.8 (11)
O1—Tb1—N2—C10	43.9 (5)	O2—C26—C27—C28	25.8 (14)
O4—Tb1—N2—C10	−163.0 (6)	C25—C26—C27—C28	−154.6 (10)
O6—Tb1—N2—C10	−106.6 (6)	O2—C26—C27—C32	−154.9 (10)
N1—Tb1—N2—C10	176.8 (6)	C25—C26—C27—C32	24.8 (15)
O3—Tb1—O2—C26	44.0 (9)	C32—C27—C28—C29	−1.3 (17)
O5—Tb1—O2—C26	−13.2 (12)	C26—C27—C28—C29	178.0 (11)
O1—Tb1—O2—C26	−34.6 (9)	C27—C28—C29—C30	0.4 (19)
O4—Tb1—O2—C26	117.4 (9)	C28—C29—C30—C31	2(2)
O6—Tb1—O2—C26	169.1 (9)	C29—C30—C31—C32	−2.8 (19)
N2—Tb1—O2—C26	−104.5 (9)	C28—C27—C32—C31	0.2 (18)
N1—Tb1—O2—C26	−160.0 (10)	C26—C27—C32—C31	−179.2 (11)
C1—C2—C3—C4	2(3)	C30—C31—C32—C27	2(2)
O2—Tb1—O3—C33	43.7 (7)	Tb1—O3—C33—C40	24.6 (12)
O5—Tb1—O3—C33	−165.2 (7)	Tb1—O3—C33—C34	−154.1 (6)
O1—Tb1—O3—C33	117.4 (7)	O3—C33—C34—C35	13.6 (14)
O4—Tb1—O3—C33	−34.1 (7)	C40—C33—C34—C35	−165.1 (9)
O6—Tb1—O3—C33	−103.0 (7)	O3—C33—C34—C39	−163.8 (9)
N2—Tb1—O3—C33	149.5 (6)	C40—C33—C34—C39	17.4 (14)
N1—Tb1—O3—C33	−5.1 (9)	C39—C34—C35—C36	−1.5 (17)
C2—C3—C4—C5	−2(2)	C33—C34—C35—C36	−179.0 (10)
O2—Tb1—O4—C41	−53.6 (9)	C34—C35—C36—C37	−2(2)
O3—Tb1—O4—C41	33.7 (8)	C35—C36—C37—C38	4(2)
O5—Tb1—O4—C41	95.7 (10)	C36—C37—C38—C39	−2.4 (19)
O1—Tb1—O4—C41	−10.9 (11)	C35—C34—C39—C38	3.2 (16)
O6—Tb1—O4—C41	155.2 (10)	C33—C34—C39—C38	−179.4 (9)
N2—Tb1—O4—C41	−149.2 (7)	C37—C38—C39—C34	−1.3 (17)
N1—Tb1—O4—C41	−131.3 (10)	O3—C33—C40—C41	5.3 (15)
C1—N1—C5—C4	−1.9 (17)	C34—C33—C40—C41	−176.0 (9)
Tb1—N1—C5—C4	161.9 (8)	Tb1—O4—C41—C40	−24.9 (14)
C1—N1—C5—C6	178.1 (9)	Tb1—O4—C41—C42	153.9 (6)
Tb1—N1—C5—C6	−18.0 (12)	C33—C40—C41—O4	−4.4 (16)
C3—C4—C5—N1	1.8 (17)	C33—C40—C41—C42	176.8 (8)
C3—C4—C5—C6	−178.2 (10)	O4—C41—C42—C43	18.0 (13)
O2—Tb1—O5—C48	−150.4 (10)	C40—C41—C42—C43	−163.1 (9)
O3—Tb1—O5—C48	151.0 (10)	O4—C41—C42—C47	−162.8 (10)
O1—Tb1—O5—C48	−129.3 (10)	C40—C41—C42—C47	16.1 (14)
O4—Tb1—O5—C48	91.7 (11)	C47—C42—C43—C44	−2.2 (18)
O6—Tb1—O5—C48	28.2 (10)	C41—C42—C43—C44	177.1 (12)
N2—Tb1—O5—C48	−51.4 (10)	C42—C43—C44—C45	1(2)
N1—Tb1—O5—C48	−15.2 (11)	C43—C44—C45—C46	−1(2)
C10—N2—C6—C7	2.7 (12)	C44—C45—C46—C47	2(2)
Tb1—N2—C6—C7	−157.9 (7)	C45—C46—C47—C42	−3(2)
C10—N2—C6—C5	−176.0 (7)	C43—C42—C47—C46	3.0 (16)
Tb1—N2—C6—C5	23.4 (9)	C41—C42—C47—C46	−176.2 (10)
N1—C5—C6—N2	−3.1 (12)	Tb1—O5—C48—C55	−23.3 (18)
C4—C5—C6—N2	176.9 (9)	Tb1—O5—C48—C49	157.7 (9)
N1—C5—C6—C7	178.2 (10)	O5—C48—C49—C54	158.3 (12)
C4—C5—C6—C7	−1.8 (14)	C55—C48—C49—C54	−21 (2)
O2—Tb1—O6—C56	146.4 (10)	O5—C48—C49—C50	−25.9 (19)
O3—Tb1—O6—C56	−98.2 (10)	C55—C48—C49—C50	155.0 (11)
O5—Tb1—O6—C56	−32.2 (10)	C54—C49—C50—C51	0(2)
O1—Tb1—O6—C56	2.9 (13)	C48—C49—C50—C51	−175.8 (15)
O4—Tb1—O6—C56	−162.7 (11)	C49—C50—C51—C52	−2(3)
N2—Tb1—O6—C56	50.8 (10)	C50—C51—C52—C53	3(3)
N1—Tb1—O6—C56	114.9 (11)	C51—C52—C53—C54	−1(2)
N2—C6—C7—C8	−0.9 (15)	C50—C49—C54—C53	1(2)
C5—C6—C7—C8	177.7 (9)	C48—C49—C54—C53	177.1 (13)
C6—C7—C8—C9	−3.4 (16)	C52—C53—C54—C49	−1(2)
C6—C7—C8—C11	175.9 (9)	O5—C48—C55—C56	5.6 (18)
C7—C8—C9—C10	5.5 (16)	C49—C48—C55—C56	−175.4 (11)
C11—C8—C9—C10	−173.9 (9)	Tb1—O6—C56—C55	33.0 (16)
C7—C8—C9—C14	180.0 (9)	Tb1—O6—C56—C57	−147.2 (8)
C11—C8—C9—C14	0.6 (15)	C48—C55—C56—O6	−11.1 (17)
C6—N2—C10—C9	−0.3 (12)	C48—C55—C56—C57	169.2 (10)
Tb1—N2—C10—C9	160.6 (6)	O6—C56—C57—C58	−15.3 (14)
C8—C9—C10—N2	−3.9 (14)	C55—C56—C57—C58	164.5 (10)
C14—C9—C10—N2	−178.0 (8)	O6—C56—C57—C62	164.1 (10)
C9—C8—C11—C12	0.4 (14)	C55—C56—C57—C62	−16.1 (15)
C7—C8—C11—C12	−179.0 (10)	C62—C57—C58—C59	−2.9 (19)
C8—C11—C12—C13	47.2 (12)	C56—C57—C58—C59	176.5 (12)
C8—C11—C12—C15	−50.0 (12)	C57—C58—C59—C60	1(2)
C11—C12—C13—C16	39.0 (13)	C58—C59—C60—C61	2(2)
C15—C12—C13—C16	147.8 (10)	C59—C60—C61—C62	−3(2)
C11—C12—C13—C17	167.7 (10)	C60—C61—C62—C57	1(2)
C15—C12—C13—C17	−83.6 (11)	C58—C57—C62—C61	1.9 (18)
C11—C12—C13—C14	−80.8 (10)	C56—C57—C62—C61	−177.5 (11)
C15—C12—C13—C14	27.9 (8)		
